# Distinct trajectories of perceived control over aversive stimulation predict affective reactions to stressors over and above objective control

**DOI:** 10.1038/s41598-025-19958-9

**Published:** 2025-10-07

**Authors:** Jana Meier, Laura E. Meine, Katja Schüler, Michèle Wessa

**Affiliations:** 1 Research Group Wessa, Leibniz Institute for Resilience Research, Mainz, Germany; 2 Department of Neuropsychology and Psychological Resilience Research, Central Institute of Mental Health, J5 68159 Mannheim, Germany; 3 Experimental Psychopathology and Psychotherapy, Department of Psychology, University of Zurich, Zurich, Switzerland; 4 Adult Psychiatry and Psychotherapy, Psychiatric University Clinic Zurich, University of Zurich, Zurich, Switzerland; 5 Department of Clinical Psychology and Neuropsychology, Psychological Institute, Johannes Gutenberg-University Mainz, Mainz, Germany; 6 DKFZ Hector Cancer Institute at the Medical University Center Mannheim, Heidelberg, Germany; 7 Division of Cancer Survivorship and Psychological Resilience, German Cancer Research Center (DKFZ) Heidelberg, Heidelberg, Germany; 8 German Center for Mental Health (DZPG), Partner Site Mannheim-Heidelberg-Ulm, Mannheim, Germany

**Keywords:** Learned helplessness, Controllability, Triadic design, Immunization, Psychology, Human behaviour

## Abstract

Psychological theories and evidence from animal and human studies highlight the importance of stressor controllability for affective stress reactions. In addition to objective control, i.e. action-outcome contingencies, higher subjective perceptions of control and trait-like control beliefs such as self-efficacy have been linked to more resilient stress outcomes. Hence, facets of perceived control may compensate for an objective lack of control. In a randomized, controlled behavioral study in healthy young adults, we studied the effect of experimentally manipulated objective control over aversive stimulation and perceived control as rated by the participants, on affective responses and tested whether a self-efficacy manipulation would buffer against the negative effects of uncontrollable stress. 168 participants were assigned to groups experiencing no (NO-STRESS), controllable (CON), uncontrollable (UNCON), or uncontrollable aversive stimulation preceded by an autobiographical self-efficacy manipulation (UNCON-HSE) while concurrently rating perceived control. The CON group reported lower helplessness and, for women, lower negative affect than the UNCON group. The self-efficacy manipulation had no effect on self-efficacy or affect. Latent class growth analysis of perceived control trajectories revealed three classes with low, rising, and medium perceived control. Compared to the rising and medium classes, the low class reported more helplessness and depression and, for women, a greater increase in negative affect. Perceived control may thus be a valuable target for resilience interventions and depression therapy.

**Supplementary Information:**

The online version contains supplementary material available at 10.1038/s41598-025-19958-9.

## Introduction

The ability to control situations with potentially adverse consequences is fundamental to maintaining mental and physical health and well-being. A substantial body of rodent research has shown that uncontrollable aversive stressors in contrast to controllable ones produce a number of behavioral, cognitive and affective impairments that have been established as a laboratory model of depression (for a comprehensive review, refer to^1^. In accordance with this, influential theories on stress and emotion, namely the transactional stress model^2^, and the component process model of emotion^3^, suggest that the appraisal of a stressor’s controllability is relevant for stress reactions and the emergence of emotions, respectively. The PASTOR model^4^ expands this concept to resilience: Appraisals of low controllability could exacerbate negative emotions and stress reactions, which in turn might increase psychopathological symptoms, while appraisals of high control could increase resilience. In particular, the impact of controllability on affective reactions might mediate resilience against stress-related disorders. Hence, animal and human models alike implicate control over stressors as a central mechanism relevant for mental health. While animal models allow for the detailed study of neural circuits, it is important to translate animal findings to humans to understand the mechanisms by which not only objective control over stressors but also its individual perception influences negative affect.

Stressor controllability research in humans has shown that control over stressors protects against a plethora of negative outcomes. Objective control over the offset or intensity of aversive stimuli was found to improve performance in instrumental^5–7^ and cognitive tasks^8–10^, executive processing^11^ and fear extinction learning^12^, to reduce activation in pain-processing^13^ and stress-related brain regions^14^, and to decrease depressed affect^15,16^. These findings are complemented by studies manipulating the perception of control, rather than actual control conditions. Perceived control was related to increased persistence after setbacks in an academic course game^17^, reduced posttraumatic distress^18^ and decreased neural pain processing and perceived pain intensity^19,20^. Moreover, perceived but not objective control was related to better working memory performance and associated neural processing^21^. Hence, human studies provide evidence that control over aversive stimuli exerts a protective effect on cognitive, motivational, and neural functioning. Moreover, they indicate that the perception of control might be equally or even more relevant than objective control for successful stress coping. While manipulations of objective control are more comparable to rodent studies, perceived control may represent a valuable target for mental health interventions in humans.

Indeed, the effectiveness of cognitive-behavioral therapy in reducing depression may be mediated by changing individuals’ expectations about the controllability of negative life events. For example, the hopelessness theory of depression posits that hopelessness, i.e. the expectation that negative outcomes will occur and there is nothing that one can do about it, is the proximal cause for depression^22^. It further states that hopelessness is the result of negative inferential style in the presence of negative life events and that therapy should focus on changing this inferential style. This is supported by some putative evidence that intervention aimed at correcting negative inferential style reduce depression symptoms and dysphoria^23^. Negative inferential style is described as inferring stable and global causes for negative events, and inferring negative consequences and negative self-characteristics from negative events^22^. Interventions that emphasize personal control over negative life events could reduce dysfunctional inferential styles and may be suitable to prevent psychopathology and increase resilience.

Individuals hold generalized beliefs about their ability to control important aspects of their lives that are related to mental health^24^. Bandura’s^25^ concept of self-efficacy, the expectation that one is able to perform an action that leads to a desired outcome, may represent such a trait-like control belief. General self-efficacy has been shown to predict good mental health and reduced psychological distress and depression in adolescents, college students and older adults^26–28^. Increasing self-efficacy through psychological interventions might thus provide a possibility of primary prevention against psychopathology^29^. For instance, giving participants positive false feedback concerning their allegedly superior coping skills increased their self-efficacious self-description and social problem solving^30^. A recent paper reported that a smartphone-based self-efficacy intervention encompassing the recall of personal mastery experiences decreased hopelessness and trait anxiety in university students with elevated stress levels^31^, suggesting that self-efficacy could be improved with brief interventions. In situations where objective control is absent or not observable, self-efficacy might preserve proactive coping^32^. A better understanding of the relative impact and the interaction of objective and perceived control, as well as control beliefs on stress processing might thus improve our understanding of resilience and inform intervention development.

The goal of this study was thus to investigate the interplay of objective control and perceived control on affective reactions to stressors and test a putative buffering effect of a brief self-efficacy enhancing manipulation. We employed a task manipulating control over stressors recently developed by Meine et al.^7^ that closely follows rodent experiments. We compared a group experiencing controllable aversive stimulation (CON) to a group experiencing yoked uncontrollable stimulation (UNCON) and a no-stress control group (NO-STRESS). For the investigation of a buffering effect of self-efficacy, half of the UNCON group underwent a self-efficacy enhancing manipulation consisting of fake feedback on coping skills and recall of self-efficacious memories before undergoing uncontrollable aversive stimulation (UNCON-HSE). NO-STRESS, CON and the other half of the UNCON group completed a neutral condition instead (NoHSE). The analyses on perceived control were based on a rating of subjective control that was obtained at five time points throughout the experiment. While others have performed a median split to obtain groups with low and high perceived control from similar ratings^21^, we used growth mixture modeling (GMM). This data-driven method allowed us to extract classes of trajectories from the time series data without information loss. We investigated whether the classes derived from observed perceived control explained differences in affect over and above the objective control. Because findings from rodent studies on stressor controllability have recently pointed at substantial sex differences^33^, we recruited a sample counterbalanced for gender and included gender in all analyses. We hypothesized that objective control over stressors would protect against stress-related impairments in affect, i.e. there would be an increase in helplessness and negative affect in response to a stressor controllability task in the groups experiencing aversive stimulation, compared to the NO-STRESS groups, but this increase would be reduced in the group experiencing control over stressor offset (CON), compared to the groups with no control (UNCON). Further, we expected that a brief manipulation designed to enhance self-efficacy would buffer the effect of uncontrollable stress, resulting in lower helplessness and negative affect in the UNCON-HSE, compared to the UNCON group. Finally, we expected perceived control to have similar protective effects as objective control. Due to the exploratory nature of GMM, we could not predict the number of latent perceived control trajectories that we would observe but hypothesized that classes with higher perceived control would show lower negative affect.

## Methods

The study was registered as a clinical trial at the German Register for Clinical Studies (DRKS; trial ID DRKS00036183) on 13.03.2025. It was conducted in accordance with the declaration of Helsinki and was approved by the ethics committee of the psychological institute of the Johannes Gutenberg University Mainz (2016_JGU_psychEK_007). Data collection took part from September 2018 to November 2019 at Johannes Gutenberg University Mainz, Germany. All participants provided written informed consent prior to data acquisition.

### Participants

A power analysis conducted in G-Power 3.1.9.7, based on a pilot study^7^ where we found an effect of perceived control on escape efficiency, η² = 0.12, indicated that a sample of *N* = 84 would be sufficient to detect an effect of that size with a power of 0.80 and an alpha level of 0.05. However, as we added the self-efficacy manipulation and had no a priori information about the interaction with the controllability effect, we based power calculation and accordingly sample size on a medium effect size. Results of power analysis revealed that a sample size of *N* = 179 would be sufficient to find a medium sized (f = 0.25) group difference with a power of 0.80 at an alpha level of 0.05. We were able to recruit 175 healthy young adults (50% female) aged between 19 and 30 years (*M* = 24.07, *SD* = 3.33). While the sample included psychology and medical students, additional participants were recruited via mailing lists, social media and flyers and contact data obtained from the local residents’ registration office in order to achieve a more representative sample. In the final sample, 103 participants were students, 3 of which were compensated with course credit while the other participants received 50€ for their participation. All participants were screened prior to data acquisition. We excluded individuals with a current or lifetime psychological diagnosis, assessed with a custom adaptation of the Structured Clinical Interview for DSM-IV axis I disorders (SCID-I), as the study was intended as proof-of-principle of the translational stressor controllability task and we were interested in basic processes related to controllability in healthy individuals. Many mental illnesses, including depression, tend to reoccur after recovery and it has been hypothesized that one episode of illness may leave traces in the brain that facilitate later episodes^34^. We further excluded individuals with certain physical conditions (e.g. migraine, due to the task being a potential trigger), medication that could influence stress processing or affect, a BMI below 18.5 or above 26 as higher BMI has been associated with changes in stress processing (Verdejo-Garcia et al., 2015), insufficient German language skills, tinnitus or hearing problems, skin problems at the site of electrode attachment, dyslexia as it may interfere with the reading span task that was conducted, and left-handedness, as the electrode had to be attached to the left hand for logistic reasons. Two participants were not willing to continue the study after the first appointment. Of the remaining 173 participants who completed both testing days, five were excluded due to receiving wrong instructions (two in NO-STRESS group), no ECG recording (one in CON group) or data saving problems (two in NO-STRESS group). The final sample consisted of 168 participants.

### Procedure

After screening, participants were invited to two appointments not more than two weeks apart. They were randomly assigned to four groups, stratified for gender. The study comprised two experimental manipulations: a self-efficacy manipulation with two conditions (high-self efficacy and a neutral control condition) and a stressor controllability task with three conditions (a controllable stress, a yoked uncontrollable stress and no-stress control group). We did not employ a fully crossed design as we mainly sought to investigate whether a boost in perceived self-efficacy would buffer against detrimental effects of uncontrollable stress. Therefore, one group experienced the high self-efficacy condition followed by uncontrollable stress (UNCON-HSE), whereas the remaining three groups underwent a neutral condition (noHSE) before experiencing controllable (CON), yoked uncontrollable (UNCON) or no stress (NO-STRESS). The CON group comprised 50 participants, the UNCON and UNCON-HSE groups 40 participants each and the NO-STRESS group 38.

On the first testing day, baseline measures of cognitive abilities, hair cortisol, demographic data, trait anxiety and depression were acquired, as well as other trait questionnaires that were part of the central project of the Collaborative Research Center 1193 but are not further reported here. At the second appointment, participants first received basic information about the upcoming procedures before we measured baseline affective state and reaction time. Next, participants were informed about the aversive stimulation and the electrical stimulus was calibrated individually. Then, participants underwent the self-efficacy manipulation and the stressor controllability task. Next, participants again completed self-report measures of affect, as well as an escape behavior task^7^ and a reading span task assessing working memory^35^. The present article focuses on affective outcomes of control over stressors.

### Self-efficacy manipulation

We aimed to experimentally enhance perceived self-efficacy expectancy in the UNCON-HSE group. To that aim, we delivered false feedback concerning coping skills, plus an autobiographical memory task which in the remainder of the article will be called high-self-efficacy condition. The recall of self-efficacious memories has been shown to reduce hopelessness and anxiety^31^. We chose to combine it with the false feedback on good coping skills as this has been found to enhance the specificity, positive content and self-efficacy content of autobiographical memories^30^ and we thus deemed this combination as especially effective in increasing self-efficacy. Participants in the high self-efficacy condition received false feedback regarding their coping skills which they were told was based on the questionnaire data they provided on the first testing day. They were told that they had achieved a percentile rank of 91 relative to a comparison group and thus belonged to the 10% who most efficiently deal with negative live events. We emphasized that the study aimed to identify processes that allow successful coping in difficult situations and asked them to describe three situations that they had handled successfully, as well as their personal strategies and strengths that enabled them to do so. The CON, UNCON and NO-STRESS groups underwent a neutral control condition (noHSE) equal in duration to the HSE condition. They received no false feedback on their coping skills, but a neutral overview of the questionnaires they had completed. They were then asked to describe three everyday routines, like getting ready for work, as well as their objective and frequency. Changes in participants’ assessment of their self-efficacy expectancy was measured before and after the manipulation, with the item “When you think about the future: how do you evaluate your ability to cope with upcoming stressful situations?” rated on a 5-point Likert scale ranging from *very bad* to *very good.*

### Aversive stimulation

The aversive stimuli during the stressor controllability task consisted of concurrent 100ms white noise bursts at 85dB and electrical shocks applied through an electrode attached to the back of the non-dominant hand. The noise was presented via Sennheiser HD 380 Pro headphones and the electrical stimuli were produced by a Digitimer DS7 A direct current stimulator. The intensity of the electrical stimulus was calibrated individually for each participant. The stimulus intensity was rated on a visual analog scale of 10 cm with 4 anchors (*barely perceptible*,* unpleasant*,* very unpleasant* and *really painful*). After determining the perceptual threshold, shock intensity was increased by 0.5 mA until a rating of *unpleasant* was reached. Participants were not informed what the target rating was, to avoid false ratings and they were not able to see the device controlling the current intensity. Moreover, experimenters emphasized that the perceived intensity could vary individually and participants should also report when their perception decreased or stayed the same despite an increase in amperage. The calibration procedure was performed in the same way for all experimental groups, even though the NO-STRESS group did not experience aversive stimulation during the stressor controllability task.

### Stressor controllability task

**Fig. 1:**
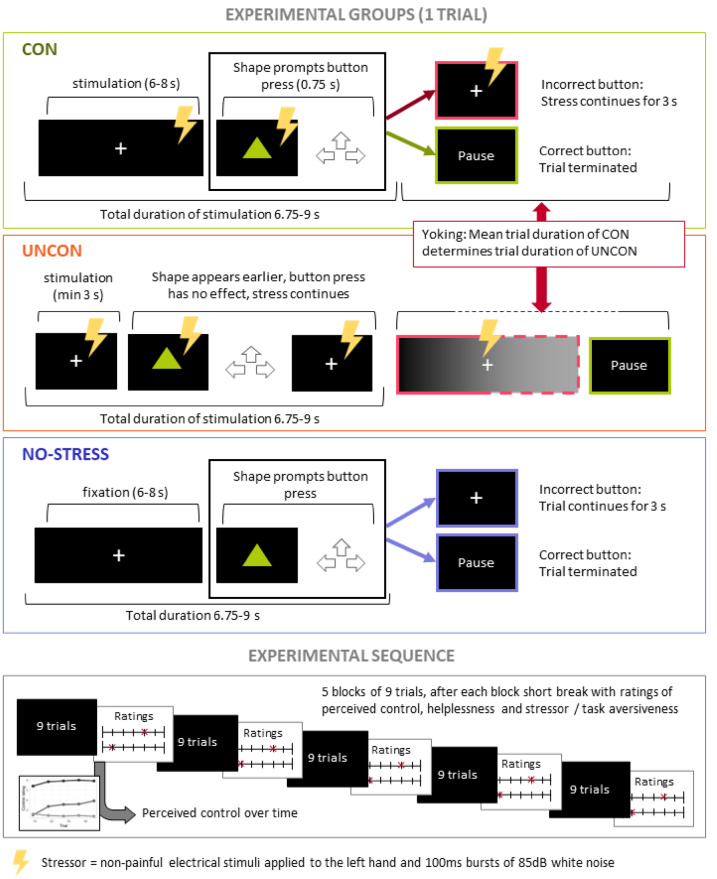
Stressor controllability task. *Note.* Sequence of one trial in each experimental group and task sequence for all groups. Trials started with a fixation period, for CON and UNCON paired with aversive stimulation, followed by the presentation of one of 3 geometric shapes. CON and NO-STRESS participants could terminate trials by pressing the correct arrow key paired with this shape upon shape presentation. Key presses had no effect for UNCON participants, trial durations for UNCON were yoked to CON participants. Participants underwent 45 trials in 5 blocks and rated perceived control, helplessness and task/stressor aversiveness on 7-point Likert scales after each block.

Subsequently, a task manipulating control over stressful stimuli, adapted from Meine et al.^7^, was employed. Figure 1 gives a graphical and schematic overview of the experimental design. Participants completed one of three conditions. Trials for the CON group started with a white fixation cross on a black screen. After 1s, stressful stimulation began and participants received concurrent double shocks 20ms apart, every 1.5s +/-0.25s and white noise bursts divided by short breaks of 50-200ms. After 8s +/- 0.25s of aversive stimulation, a green triangle, a square or a circle were presented on screen. Participants had to press either the arrow upwards, to the left or to the right within 0.75s. Each key was assigned to one shape and participants had to figure out the correct assignment through trial and error. When the correct key was pressed in time, the trial ended and an inter-trial interval of 3s +/- 0.25s followed, during which the words “short break” were displayed. When a wrong key or no key was pressed in time, the fixation cross reappeared and the aversive stimulation continued for another 4s before the inter-trial interval started. To increase the perception of control, the time (initially 8s) that passed until the shape was presented was reduced over the course of the experiment by 0.5s after every 9th trial to 6 s in the last block.

For the UNCON and UNCON-HSE groups, key presses had no effect on the termination of the aversive stimulation. Instead, the length of each trial was based on the duration of the respective trial in the CON group. To that aim, the 50 CON participants were split into five equally sized groups. Within these groups, the mean duration for each trial across participants was computed, resulting in five yoking files. Trial durations in the UNCON group were then set to these mean durations, with the yoking file counterbalanced across the UNCON participants. To increase the perception of uncontrollability and prevent the illusion of control due to increasingly shorter trial durations over the course of the experiment, the onset of the shape was shifted forward in time for the UNCON participants. This ensured that even if all the yoked CON participants had pressed the correct button, the stressor would not stop immediately after the shape presentation but instead, the shape appeared earlier for the UNCON participant, and the stressor continued for a few seconds. This time shift of the shape was uniformly distributed between 0 and − 4.75s with respect to the shape presentation in the CON group.

For the no-stress control condition (NO-STRESS), trials were identical to the CON condition, but participants received no aversive stimulation. They were told to press one of the arrow keys as fast as possible once the shape appeared.

All CON, UNCON and UNCON-HSE participants were informed that correct keypresses would terminate the stressful stimulation, though this was only true for the CON group. Participants underwent a total of 45 trials. After every 9th trial, they rated their perceived control and helplessness, as well as the aversiveness of the stressor (stress groups) or the task (NO-STRESS group) on a 7-point Likert scale ranging from *not at all* to *very*.

### Self-report measures

In addition to the ratings of self-efficacy, perceived control, helplessness and aversiveness during the experimental manipulations, participants completed self-report questionnaires. Prior to the self-efficacy manipulation, we assessed generalized self-efficacy with the German General Self-Efficacy Scale (GSE)^36^ that comprises ten items rated on a 4-point Likert scale ranging from *not at all true* to *exactly true.* The values were summed up to a score ranging between 10 and 40, with higher values indicating greater general self-efficacy expectancy. The GSE was found to have acceptable to excellent reliability (Cronbach’s α = 0.76 – 0.90) in samples from 23 nations^37^, and has been shown to be a valid measure of self-efficacy that predicts health-related behaviors, as well as anxiety, depression, and quality of life in different cultures^38^.

Before and after the stressor controllability task, participants completed questionnaires concerning their affective state: Depression and anxiety were assessed with the state version of the State-Trait Anxiety and Depression Inventory (STADI-S; 39). In the STADI-S, depression and anxiety are differentiated with a 4-factor model that has been confirmed by factor analysis^40^. The subscales have good reliability, Cronbach’s α = 0.90 for anxiety and α = 0.87 for depression^39^. It contains 20 items rated on a 4-point Likert scale from *not at all* to *very*. Inverted items were recoded so that higher scores indicated higher symptoms and subscales for anxiety and depression were computed. The STADI is an adaptation of the well-established state-trait anxiety inventory (STAI;^41^ that aims to better differentiate anxiety and depression components. The STADI correlates in the expected way with other measures of depression and anxiety, scores have been shown to reflect worsening of symptoms before stressful live events (examinations) and improve during hospitalization and investigations in healthy and clinical samples indicate good convergent and discriminant validity^39,40^. Negative affect was assessed with the Positive and Negative Affect Schedule (PANAS; 42) that consists of 20 adjectives. Participants rated on a 5-point Likert Scale ranging from *not at all* to *extremely* to what extent they felt in the described way at that moment. Internal consistency for the PANAS is good, α = 0.86, and the factor structure with 2 distinct factors representing positive and negative affectivity has been confirmed^42^.

### Analysis

All statistical analyses were performed in R 4.2.2^43^ running in RStudio 2023.12.0.369^44^. For state anxiety, depression and negative affect, 2 participants (1 NO-STRESS, 1 CON) had missing values and were excluded from the analysis for these outcomes. We tested for baseline group differences in age, performance in the stressor controllability task and general self-efficacy with one-way ANOVAs with the factor group (CON, UNCON, UNCON-HSE, NO-STRESS). As the representativeness of college students for the general population is questionable, we compared students to non-students in all outcome measures with *t*-tests. To check the success of the self-efficacy manipulation, we conducted a mixed ANOVA using the R package afex^45^ for the self-efficacy rating with the within-subject factor time point (pre, post) and the between-subject factors self-efficacy condition (HSE, noHSE) and gender (male, female).

For the between-group analyses of the effects of the self-efficacy manipulation and the stressor controllability task, we conducted two-way ANOVAs with the factors group and gender and the interaction between the two for task/stressor aversiveness, mean perceived control, depression, anxiety and negative affect. For all analyses involving the factor group, planned orthogonal contrasts compared (1) the NO-STRESS group to the stress groups, (2) the CON to the UNCON groups, and (3) the UNCON to the UNCON-HSE group. In the case of significant interactions, post-hoc tests were computed with the emmeans package for the relevant comparisons using Tukey correction for multiple testing^46^.

#### Growth mixture modeling of perceived control

For the analysis of subjective control, we excluded the 38 NO-STRESS participants because they had not experienced any stressors, resulting in a sample size of *N* = 130. Then, using the lcmm package^47^, we performed growth mixture modeling (GMM) on the perceived control ratings that had been assessed at five timepoints over the course of the stressor controllability task. We first fitted growth mixture models with random intercepts only and then with random intercepts and slopes. This allows the intercepts and slopes to vary within classes, which can help to identify relevant heterogeneity in the data without resulting in too many small classes. The variance-covariance matrix of the random effects was freely estimated and allowed to vary between classes. This is in line with recommendations to fit flexible models where constraints on the parameters are relaxed, which may be better suited to identify relevant classes and avoid over-extraction^48^. All models were fitted using 500 sets of random start values run for a maximum of 10 iterations and then only the estimation with the best log-likelihood was finalized to avoid local solutions^49^. For selection of the best model, it is recommended to consider multiple criteria rather than one single fit statistic^50^. We therefore chose the best fitting model based on fit indices, discriminatory power and theoretical plausibility. For the Bayesian Information Criterion (BIC), smaller values suggest better model fit. Entropy (the better the closer to 1) provides information on discriminatory power. Entropy greater than 0.7 reflects acceptable classification and classes that contain less than 1% of the participants are considered too small to reflect relevant heterogeneity^51^. However, due to our relatively small sample size, we considered classes with less than 10% of the sample as too small, because that would correspond to less than 13 participants. Such a small class is unlikely to represent a relevant subsample and it would have too little statistical power for between-class analyses.

Subsequently, we tested whether the obtained classes of perceived control predicted stress outcomes over and above the objective control conditions. To that aim, we conducted ANOVAs for negative affect, depression and anxiety with class and gender as between-subject factors and included the objective controllability condition (CON or UNCON) as covariate to control for effects of the original experimental condition in the stressor controllability paradigm. Post-hoc tests were computed using the emmeans package with the Tukey method to correct for multiple comparisons^46^.

Prior to conducting ANOVAs, we assessed whether the assumptions of the statistical procedures were met via a priori tests and visual inspection. The assumption of normality was violated for nearly all variables. Additionally, variances were unequal between conditions for perceived helplessness (Levene test: *p* < 0.001) and negative affect (*p* = 0.041). However, ANOVA is rather robust against even severe violations of the assumption of normality^52^, especially for bigger sample sizes. The result of unequal variances was mostly driven by the very low variance in the NO-STRESS group for subjective helplessness and negative affect. The NO-STRESS group also had the smallest sample size. ANOVA is not robust against variance inhomogeneity in unbalanced designs: When sample size and variances are proportional, i.e. groups with bigger sample sizes have bigger variances, power decreases^53^. Therefore, results on subjective helplessness and negative affect might be too conservative, but a type I error is unlikely. Moreover, sample sizes were not substantially different with the n ranging from 18 to 26.

All dependent variables were checked for outliers using the Rstatix package and values above or below three times the interquartile range were considered extreme outliers and excluded from analyses^54^. For the self-efficacy rating, 57 data points were considered extreme outliers due to the interquartile range being 0 for some time points. For feasibility reasons, we did not perform outlier exclusion for this measure. For the comparisons between the objective control groups, we excluded 3 participants for the key press performance (all NO-STRESS), 3 participants for subjective helplessness (all NO-STRESS), 1 participant for state depression (UNCON), 1 participant for state anxiety (NO-STRESS), and 11 participants for negative affect (6 NO-STRESS, 1 UNCON-HSE, 4 CON). For the comparisons between the perceived control classes, outlier handling resulted in the exclusion of 1 participant for state depression (medium class), 2 participants for state anxiety (rising class) and 6 participants for negative affect (4 low, 1 rising, 1 medium class). Moreover, 2 participants had to be excluded from the analyses for state depression, anxiety and negative affect due to missing data.

The alpha-level was set to 0.05. We Holm-corrected the p-values for the main effects of group and class and the interaction effects with gender for the number of outcome variables. For all ANOVAs, we used type II sums of squares as recommended by Venables^55^. We report eta squared (η²) as effect size because it is more easily interpretable than partial eta squared^56^. For the mixed ANOVA, we report generalized eta squared (η²_g_)^57^.

## Results

### Baseline and manipulation checks

Statistics on baseline and manipulation checks between the groups can be found in Table 1. None of the groups differed in age, gender or general self-efficacy. *T*-tests comparing students and non-students in performance in the stressor controllability task, perceived stressor aversiveness, control, helplessness, as well as general self-efficacy, state depression and anxiety, and negative mood showed no significant differences, all *p*s > 0.05.

**Table 1:** Descriptive statistics for the experimental groups.

Variable	CON	UNCON	UNCON-HSE	NO-STRESS	Full Sample	F	*p*
Sample Characteristics							
N	50 (29.8%)	40 (23.8%)	40 (23.8%)	38 (22.6%)	168 (100%)		
Female	24 (48%)	20 (50%)	20 (50%)	20 (52.6%)	84 (50%)		
Male	26 (52%)	20 (50%)	20 (50%)	18 (47.4%)	84 (50%)		
Age	24.24 (3.19)	24.73 (3.73)	23.58 (3.33)	23.66 (3.05)	24.07 (3.33)	1.03	0.373
Highest educational degree							
None	-	-	1	-	1		
Middle school	2	1	-	-	3		
High school	25	19	22	25	91		
Apprenticeship	7	9	2	2	20		
University degree	16	11	15	11	53		
General self-efficacy	30.26 (3.65)	31.40 (4.21)	30.85 (4.40)	30.16 (3.28)	30.65 (3.90)	0.90	0.445
Stressor Controllability Task							
Stressor / task aversiveness	5.24^a^ (1.33)	4.91^a^ (1.39)	5.27^a^ (1.12)	1.82^b^ (1.10)		70.81	< 0.001
Perceived control	3.39 (1.52)^a^	2.67 (1.55)^b^	2.21 (1.30)^b^	5.82 (1.28)^c^	3.49 (1.94)	51.61	< 0.001
% correct key presses	53.11^a^ (20.38)	18.11^b^ (9.49)	21.50^b^ (11.70)	31.87^c^ (5.45)		60.19	< 0.001
Stress phase duration^1^	9.18 (0.70)^a^	9.15 (0.14)^a^	9.15 (0.14)^a^	9.87 (0.20)^b^	9.32 (0.50)	29.36	< 0.001

Note. Groups did not differ in age, gender and general self-efficacy. Aversiveness ratings were higher for the stress groups, compared to NO-STRESS. Perceived control and correct key presses indicate successful manipulation of stressor controllability. Duration of aversive stimulation did not differ between controllable (CON) and uncontrollable (UNCON) groups.

For continuous variables: Mean (SD). For categorical variables: Count (Percent). Different superscripts indicate that groups significantly differ from each other. *P*-values indicate effect of condition in ANOVA or Welch test.

^1^ In NO-STRESS group, this phase included no stressful stimulation.

#### Stressor controllability task

**Fig. 2:**
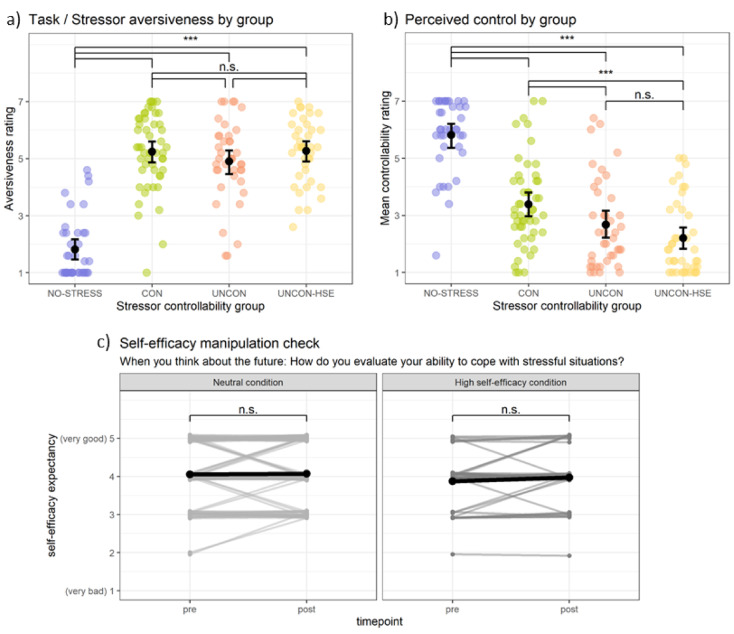
Manipulation checks. *Note.* (**a**) Task / stressor aversiveness was higher in the stress, compared to the NO-STRESS group, indicating successful stress induction. (**b**) Perceived control was highest in the NO-STRESS, followed by the CON group and lowest in the UNCON groups, reflecting objective control. (**c**) Self-efficacy rating before and after the self-efficacy intervention for the HSE group (right) and the noHSE groups (left). The intervention did not increase self-efficacy in any group.

CON participants performed above chance level while UNCON and NO-STRESS participants had no possibility to figure out the correct response and performed at or below chance level. The stress groups rated the task/stressor as significantly more aversive than the NO-STRESS group (See Fig. 2a). The perceived control rating shows effective manipulation of control: It was highest in the NO-STRESS group, followed by the CON group and finally the UNCON and UNCON-HSE groups, which did not differ significantly (see Fig. 2b). The mean trial duration, which corresponds to the duration of aversive stimulation in the stress groups, was longer in the NO-STRESS group but did not differ between the CON and UNCON groups, indicating successful yoking of stressful stimulation. Gender differences were also observed: Women rated the task/stressor as significantly more aversive *F*(1,160) = 18.36, *p <* 0.001 and perceived significantly less control than men, *F*(1,160) = 10.25, *p* = 0.002, η²=0.03.

#### Self-efficacy manipulation

The 2 × 2 × 2 mixed ANOVA revealed no significant effect for self-efficacy condition, *F*(1,164) = 1.37, *p* = 0.243, η²_g_=0.008 and time, *F*(1,164) = 2.68, *p* = 0.103, η²_g_=0.001, but a significant effect of gender, *F*(1,164) = 4.66, *p* = 0.032, η²_g_=0.025 and a significant interaction of gender and time, *F*(1,164) = 4.33, *p* = 0.039, η²_g_=0.002. The remaining interactions did not reach significance, *p*s > 0.2. Participants reported very little change in self-efficacy ratings in general (see Fig. 2c). Post-hoc tests revealed that women’s self-efficacy ratings increased from pre to post, while men’s did not. However, there was no significant difference between the conditions, so the change was not attributable to the manipulation. Thus, the self-efficacy manipulation had no effect on participants’ self-efficacy ratings. Moreover, we did not observe any difference between the UNCON and HSE-UNCON groups in any outcome (for details, please refer to the supplementary Table S1). For the sake of additional power, we collapsed the UNCON and UNCON-HSE groups for further analyses.

### Results on objective controllability and affect

Summary statistics and inferential statistics for the group comparisons in the affective outcomes can be found in supplementary Table S2.

#### Perceived helplessness

**Fig. 3:**
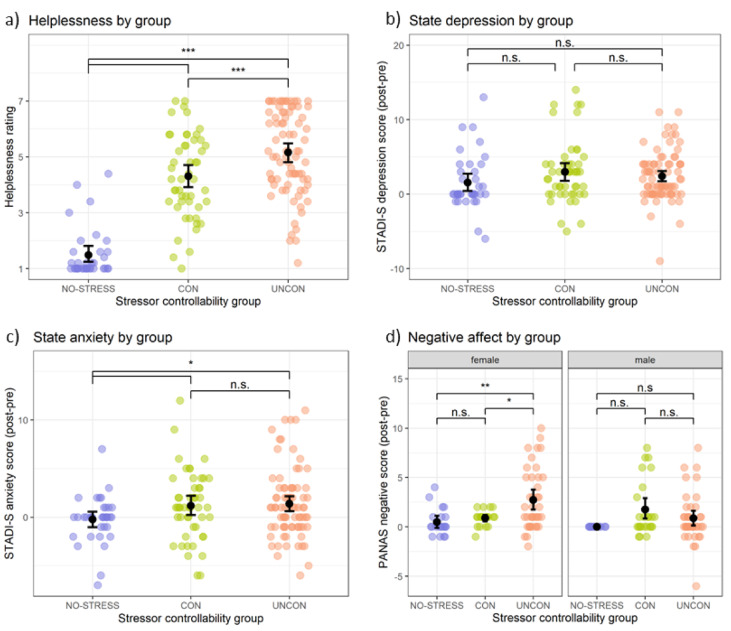
Effects of objective stressor controllability on affect. *Note.* Differences between the controllable (CON), uncontrollable (UNCON) and no-stress control (NO-STRESS) groups in (**a**) mean perceived helplessness during the stressor controllability task, change in (**b**) state depression, (**c**) state anxiety, and (**d**) negative affect from before to after the task. Objective control reduced helplessness and, for women, negative affect. Error bars denote bootstrapped 95% confidence intervals for the means. STADI-S = State version of the State-Trait-Anxiety-Depression-Inventory, PANAS = Positive and Negative Affect Schedule, n.s. = non-significant, *p < 0.05, **p < 0.01, ***p < 0.001.

The groups differed in their perceived helplessness during the stressor controllability task, *F*(2,159) = 88.47, *p*_*adj*_<0.001, η²=0.511 (see Fig. 3a). Planned comparisons revealed that the CON group reported less helplessness than the UNCON group, but more than the NO-STRESS group, all *p*s_adj_<0.001. A significant gender effect showed that, in general, women reported more helplessness than men, *F*(1,159) = 6.36, *p* = 0.013, η²=0.019. The interaction group x gender was not significant, *F*(2,159) = 0.24, *p*_*adj*_>0.999, η²=0.001.

#### Depression and anxiety

There was no significant effect of group, *F*(2,159) = 1.84, *p*_*adj*_=0.163, η²=0.020, or interaction group x gender, *F*(2,159) = 0.80, *p*_*adj*_>0.999, η²=0.009, on state depression (see Fig. 3b) but a significant effect of gender, *F*(1,159) = 6.42, *p* = 0.012, η²=0.038 with women reporting a stronger increase in depression over the course of the experiment. For the change in state anxiety, the effect of group approached significance, *F*(2,159) = 3.13, *p*_*adj*_=0.093, η²=0.036. Contrasts revealed that the NO-STRESS group reported less increase in anxiety than both stress groups, *p*_*adj*_=0.018, while the CON and UNCON group did not differ from each other, *p*_*adj*_=0.756 (see Fig. 3c). No significant gender (*F*(1,159) = 2.99, *p* = 0.086, η²=0.018) or interaction effects (*F*(2,159) = 0.65, *p*_*adj*_>0.999, η²=0.008) were observed.

#### Negative affect

The effects for group, *F*(2,149) = 4.91, *p*_*adj*_=0.026, η²=0.050, gender, *F*(1,159) = 4.29, *p* = 0.040, η²=0.025, and the interaction group x gender, *F*(2,159) = 4.63, *p*_*adj*_=0.045, η²=0.054, reached significance (see Fig. 3d). Post-hoc tests revealed that in women, the UNCON group reported a stronger increase in negative affect than the CON (*p*_*adj*_=0.017) and NO-STRESS (*p*_*adj*_=0.003) groups. For this outcome, removing statistical outliers changed the results of the ANOVA. For the results with statistical outliers not removed, please refer to the supplementary Figure S1 and Table S3.

### Growth mixture modelling results: perceived control trajectories

**Fig. 4:**
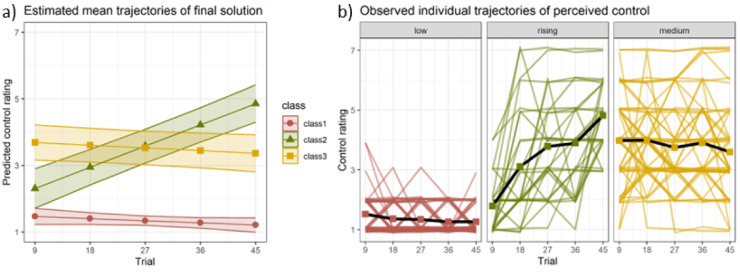
Results of GMM. *Note.* (**a**) Estimated and (**b**) observed trajectories of perceived control for the chosen growth mixture model with three latent classes and random intercepts. Classes followed on average low, rising and medium trajectories, respectively.

For the analysis of perceived control, we first fitted models with random intercepts only and 1–6 classes and compared the model fit (see Table 2). The model with the lowest BIC was the 3-class model; it had reasonably big classes and entropy was sufficient at 0.72. We added random slopes to the model to test whether this would improve the model fit. Indeed, the BIC of the 3-class model with random slopes was lower and entropy was higher. However, the slopes-model had a class made up of 6.9% of the sample which we considered too small. Therefore, we continued with the 3-class model with random intercepts only. For this selected model, the trajectories of perceived control of the respective classes can be seen in Fig. 4. Only class 2 had a significant coefficient for the slope *b* = 0.07, *p* < 0.001, which confirms the visual impression that this class had a rising trajectory while the other two classes remained stable over time, albeit at different levels. We termed the classes low, rising and medium, according to their general trajectory. Class assignment did not differ between students and non-students, as confirmed by a non-significant χ²-test, χ(2) = 1.39, *p* = 0.499.

**Table 2:** Model comparison for GMM.

Name	G	Max LL	con	npm	BIC	Entropy	% participants in classes					
							1	2	3	4	5	6
1 int	1	-1050.7	1	4	2120.9	1.00	100					
2 int	2	-1020.5	1	8	2079.9	0.80	20.8	79.2				
3 int*	**3**	**-997.8**	**1**	**12**	**2054.0**	**0.72**	**38.5**	**21.5**	**40.0**			
3 int + slope	3	-988.2	1	14	2044.6	0.86	6.9	37.7	55.4			
4 int	4	-990.2	2	16	2058.2	0.78	40.8	21.5	34.6	3.1		
5 int	5	-984.8	1	20	2067.0	0.83	3.8	20.0	6.9	38.5	30.8	
6 int	6	-980.5	1	24	2077.8	0.85	6.9	17.7	16.9	34.6	3.1	20.8

Note. Model fit for different growth mixture models. The 3-class model with random intercepts only had good fit (low BIC) and entropy with reasonable class sizes.

Model formula random intercepts only: control rating ~ trial, subject = ID, random = ~ 1, mixture = ~ trial.

Model formula random intercepts and slopes: control rating ~ trial, subject = ID, random = ~ 1 + trial, mixture = ~ trial.

G = number of classes, Max LL = maximum loglikelihood, con = converged (1 = yes, 2 = no), npm = number of parameters, BIC = Bayesian Information Criterion, int = random intercepts, int + slope = random intercepts and slopes. * chosen model.

We first explored differences between the classes in the sample characteristics and parameters of the stressor controllability task. Descriptive statistics for the latent classes can be found in Table 3. The classes did not differ in age, gender, general self-efficacy or stressor aversiveness. Performance was only compared for participants in the CON group because for the other groups, no correct response existed. There was a significant difference in the performance between the classes. Participants in the low class performed significantly worse than in the medium and rising classes, *p*s < 0.001, the latter two classes did not differ, *p* = 0.151.

**Table 3:** Descriptive statistics for the latent classes.

Variable	Low	Rising	Medium	Full sample	F/ χ^2^	*p*
Sample Characteristics						
N	50 (38.46%)	28 (21.54%)	52 (40%)	130 (100%)		
Female	30 (60%)	13 (46.43%)	21 (40.38%)	64 (49.23%)	4.04	0.133
Male	20 (40%)	15 (53.57%)	31 (59.62%)	66 (50.77%)		
Age	24.22 (3.54)	24.25 (3.62)	24.12 (3.23)	24.18 (3.41)	0.02	0.982
Highest educational degree						
None	1	-	-	1		
Middle school	-	2	1	3		
High school	26	14	26	66		
Apprenticeship	6	2	10	18		
University degree	17	10	15	42		
General self-efficacy	31.04 (4.28)	30.79 (3.84)	30.56 (4.02)	30.79 (4.06)	0.18	0.837
Stressor Controllability Task						
Experimental group						
CON	8 (16%)	21 (75%)	21 (40.4%)		26.53	< 0.001
UNCON/UNCON-HSE	42 (84%)	7 (25%)	31 (59.6%)			
Stressor aversiveness	5.39 (1.42)	5.16 (1.17)	4.91 (1.19)	5.15 (1.29)	1.80	0.169
% correct key presses^1^	25.56^a^ (14.88)	53.65^b^ (18.25)	63.07^b^ (14.10)	53.11 (20.38)	15.74	< 0.001
Stimulation duration	9.30 (0.43)^a^	9.15 (0.55)	9.04 (0.36)^b^	9.16 (0.45)	4.77	0.010

Note. The latent classes derived from growth mixture modeling did not differ in gender, age, general self-efficacy or stressor aversiveness rating. Key press performance was lower and duration of the aversive stimulation longer in the low control class. CON = controllable group, UNCON/UNCON-HSE = uncontrollable group. The NO-STRESS group was excluded from perceived control analyses.

For continuous variables: Mean (SD). For categorical variables: Count (Percent). Different superscripts indicate that groups significantly differ from each other. *P*-values correspond to the effect of *class* in ANOVA, Welch or χ^2^ test.

^1^ Correct key presses are only presented for the CON group (*n* = 50) because no correct shape-key pairing existed for the other groups.

The mean duration of aversive stimulation also differed between the classes as indicated by a significant class effect. Post-hoc tests showed that participants in the low class received more stressful stimulation than the medium class, *p* = 0.007. The rising class did not differ significantly from the low (*p* = 0.333) or the medium class (*p* = 0.470). Hence, differences in affective reactions to the stressor controllability task might in part be attributable to the low class experiencing more aversive stimulation and results have to be interpreted with some caution. However, the difference was rather small with the low class receiving less than half a second of additional stimulation per trial, compared to the medium class (see Table 3).

To investigate effects of subjective control and gender on affective responses to the stressor controllability task (i.e., perceived helplessness, depression, anxiety and negative affect), we calculated 3 (class: low, rising, medium) x 2 (gender: male, female) + 2 (group: CON, UNCON) ANOVAs. We included group as a nuisance variable as class membership was not completely independent of the objective control condition: Participants in the CON-group were more likely to be classified into the rising than the low class, while the opposite was true for the UNCON-group. The probability of falling into the medium class seemed to be equal across groups. The classes did not differ in gender distribution.

#### Perceived helplessness

**Fig. 5:**
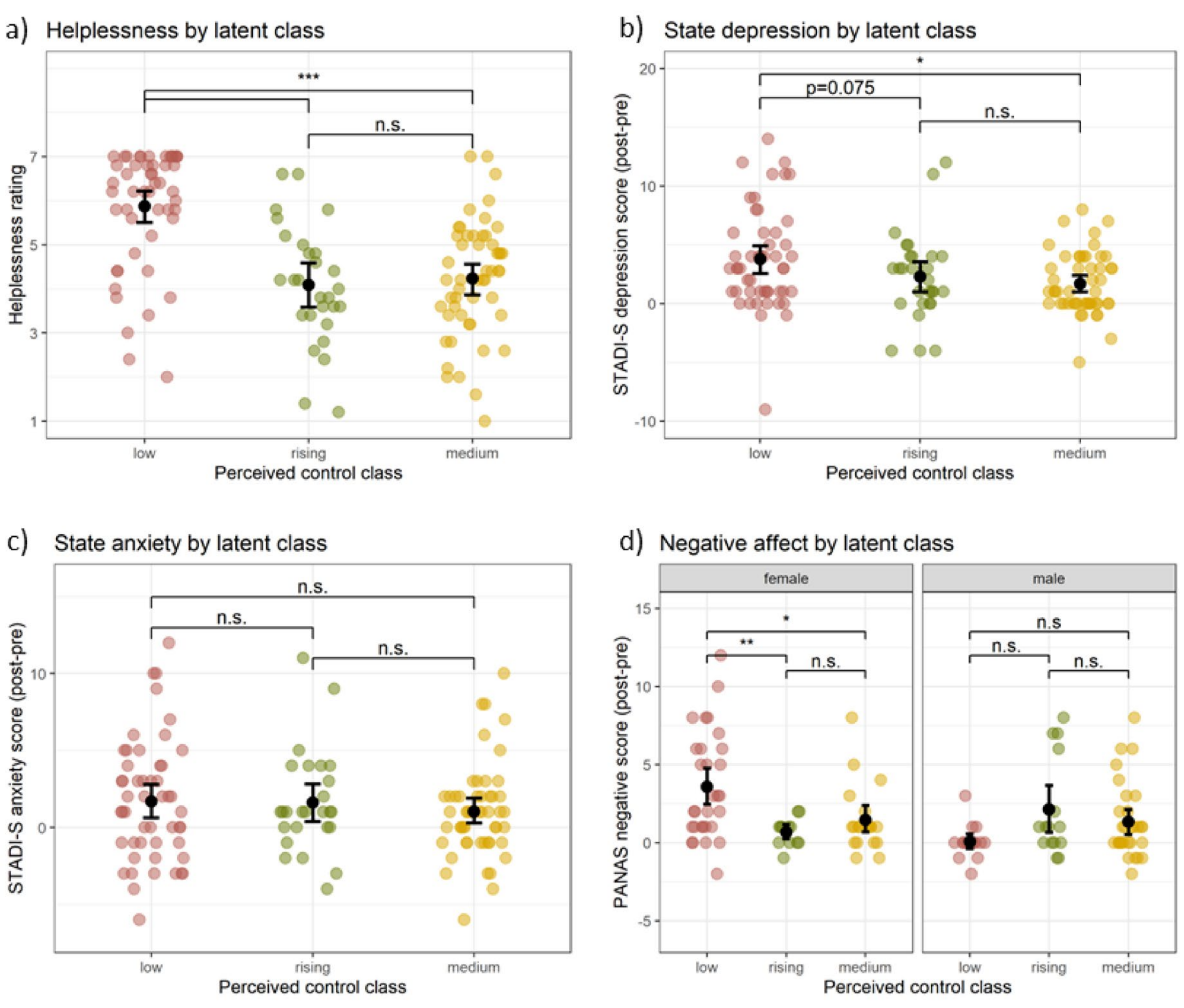
Effects of subjective stressor controllability on affect. *Note.* Differences between the latent classes with low, rising and medium perceived control trajectories in (**a**) mean perceived helplessness during the stressor controllability task, change in (**b**) state depression, (**c**) state anxiety, and (**d**) negative affect from before to after the task. The low class reported higher helplessness, a stronger increase in state depression and, for women, a stronger increase in negative affect. Error bars denote bootstrapped 95% confidence intervals for the means. STADI-S = State version of the State-Trait-Anxiety-Depression-Inventory, PANAS = Positive and Negative Affect Schedule, n.s. = non-significant, *p < 0.05, **p < 0.01, ***p < 0.001.

The ANOVA for the helplessness ratings showed a significant effect of class, *F*(2,123) = 17.28, *p*_*adj*_<0.001, η²=0.217 (see Fig. 5a). The effects for gender, *F*(1,123) = 1.13, *p* < 0.290, η²=0.007, and the interaction class x gender, *F*(2,123) = 0.16, *p*_*adj*_=0.985, η²=0.002, did not reach significance. Post-hoc tests revealed that the low class reported higher helplessness than the rising and medium classes, *p*s < 0.001, while the rising and medium class did not differ, *p* = 0.964.

#### Depression and anxiety

For state depression, we found significant main effects of class, *F*(2,121) = 4.89, *p*_*Holm*_=0.027, η²=0.090 (see Fig. 5b) and gender, *F*(1,121) = 5.40, *p*_*adj*_=0.022, η²=0.038. The interaction class x gender was not significant, *F*(2,121) = 0.71, *p*_*adjr*_=0.985, η²=0.010. Post-hoc tests showed that the low class reported a greater increase in depression than the medium class, *p*_*adj*_=0.013, and - with a trend towards significance - than the rising class, *p*_*adj*_=0.075. Women reported a greater increase in depression than men. For state anxiety, there was a significant main effect of gender, *F*(1,120) = 4.33, *p* = 0.04, η²=0.033. The main effect of class did not reach significance, *F*(2,120) = 0.35, *p*_*adj*_=0.705, η²=0.009 (see Fig. 5c), nor did the interaction class x gender, *F*(2,120) = 2.85, *p*_*adj*_=0.185, η²=0.044. Women reported more anxiety than men.

#### Negative affect

The ANOVA revealed no effect of class, *F*(2,116) = 1.20, *p*_*adj*_=0.612, η²=0.031, but a significant main effect of gender, *F*(1,116) = 4.05, *p* = 0.047, η²=0.029 and a gender x class interaction, *F*(2,116) = 8.87, *p*_*adj*_*=*0.001, η²=0.125 (see Fig. 5d). Post-hoc tests showed that women in the low class reported a significantly greater increase in negative affect than women in the rising (*p*_*adj*_=0.003) and medium class (*p*_*adj*_=0.011), while those two classes did not differ (*p*_*adj*_=0.626).

## Discussion

In this study, we investigated whether objective control over stressor offset, as well as perceived control trajectories predicted negative affective responding to that stressor, considering possible gender differences. Moreover, we aimed to explore the protective effect of a self-efficacy manipulation against impairments caused by uncontrollable stress. In sum, we found that objective, as well as perceived control over aversive stimulation decreased feelings of helplessness and, for women, also negative affect. Only perceived control was related to lower state depression. In contrast to our hypothesis, the self-efficacy manipulation had no effect on participants’ self-efficacy and did not impact affective responding to the uncontrollable stressor. The findings suggest that control over stressors can reduce stress-related increases in negative affect and individual differences in perceived control may be especially relevant for mental health-related outcomes.

Our results show that the stressor controllability task allows to study control-dependent changes in affective processing that might be related to psychopathology. Like in a previous study, control over stressors increased perceived control and decreased helplessness, and while the earlier study showed control-dependent changes in escape behavior^7^, we found gender-dependent effects of control on affective processing: For women, control reduced a stress-related increase in negative affect. Factors that reduce negative affect may be particularly important for resilience, as a negative affective processing bias is a key symptom of depression^58^. Control has been shown to modulate affective reactions to aversive noises before^16,59^ and this effect was influenced by decreasing central serotonin through tryptophan depletion^60^. This ties in with the implication of serotonin both in stressor controllability effects in rats^1^, as well as in clinical depression (^61^; although see^62^). Affective processing could then mediate the protective effect of control: Uncontrollable stress may bias affective processing negatively – possibly via circuits that are serotonin dependent, as has been proposed by the animal literature – and an antidepressant effect of control over stressors could act via improvements in emotional processing. The neural circuits underlying the effects of stressor controllability on affect and its link to depression have yet to be elucidated, but the current results indicate that the task used here may be suited for this endeavor.

In the present study, we cannot investigate the mediating role of negative affect in the relationship between stressor controllability and clinical depression, as we included only healthy participants. However, we assessed changes in state depression related to the stressor controllability task. Surprisingly, we did not observe differences in state depression between the CON and UNCON groups. Thus, reduced negative affect due to control over stressors did not result directly in reduced depressed state. However, recent research has found that perceived control might better predict stress outcomes than objective control^21^. Thus, participants’ perceptions of control might have influenced their affective reactions to the task here. This was supported by our results showing high variability in individuals’ perceived control over the course of the task. By using GMM, we identified three classes of trajectories with low, rising, and medium control perceptions. These classes differed in their affective reactions to the task: The low class reported more helplessness, a stronger increase in depressive state and, for women only, more negative affect than the rising and medium classes. Thus, perceived control predicted helplessness and negative affect like objective control but was additionally associated with state depression. We found no association of either objective or perceived control with anxiety, which is in line with the notion that stressor controllability is specifically related to depression^63^. The protective effect of perceived control on affect and depression is promising, as factual control conditions in daily life are usually difficult to modify, whereas the perceptions or appraisal of control might be improved or changed by interventions. For instance, behavioral activation (BA) therapy assumes that depression results from a lack of response-contingent positive reinforcement and employs strategies that are designed to lead to experiences of success and are likely to increase patients’ sense of control over their lives^64^. BA principles have also been implemented in cognitive behavioral therapy (CBT), the gold standard treatment for depression^64^. Our results indicate that perceived control might be a mechanism by which cognitive and behavioral therapies can reduce depressive symptoms. However, future dismantling studies particularly focusing on perceived controllability of daily stressors are necessary to shed additional light on this assumption.

Perceived control not only influenced affective reactions over and above objective control, it also seemed to buffer against an objective lack of control. Specifically, in the UNCON group, only 52.5% of participants were classified into the low class, while the other 47.5% belonged to the more beneficial rising and medium classes. Conversely, only 8 CON participants were in the low class and they also performed worse in the task, which means that they indeed had little control over the stressors despite being in the CON group. Hence, objective control was rather reliably associated with higher perceived control, but even in the absence of objective control, about half of the UNCON group still perceived some control and showed reduced affective stress responses. In rodents, differences in impairments following uncontrollable stressors have also been observed with proportions of susceptible animals varying between 14 and 30% depending on the study protocol, the parameters used and the species^65,66^. It is unclear, where these differences in susceptibility stem from. As novel and uncertain situations prompt individuals to rely on generalized control beliefs for the estimation of control^32^, we suspected that the somewhat ambiguous situation in the stressor controllability task might have facilitated the impact of individual differences in control beliefs like self-efficacy. To our surprise, general self-efficacy did not vary between perceived controllability classes. Possibly, general self-efficacy as a construct is too broad to be directly related to perceived control in a laboratory situation. A more specific construct such as measured by the coping self-efficacy scale^67^ might better explain differing control perceptions. Moreover, other factors within and outside the individual might have contributed to the differing trajectories of perceived control, ranging from previous stress experiences over genetic factors to personality traits. Future studies would therefore have to integrate different psychological and biological trait measures to elucidate on potential mechanisms underlying perceived controllability over adverse situations.

Interestingly, we found no difference in any outcome between the rising and the medium class, although the first was mostly made up of CON participants - who actually had control over the stressor - while the latter consisted to 60% of UNCON participants whose perceived control was illusory. Thus, paralleling other self-serving positivity biases that are related to mental health^68^, a slightly exaggerated perception of control might be beneficial to resilience. Future research might elucidate which factors lead individuals to perceive more control.

While BA might help individuals already suffering from depression symptoms, low-threshold interventions that enhance control beliefs might be effective for the prevention of psychopathology. We tested whether a self-efficacy manipulation would buffer against the impact of uncontrollable stress on affect. Unfortunately, the manipulation we chose did not increase participants’ self-reported self-efficacy and the HSE group did not differ from the control group in any of the stress outcome measures. In contrast to this null finding in our studies, previous systematic reviews have shown that interventions can increase general self-efficacy in patients with heart disease^69^ and addiction^70^. However, most of these interventions included direct contact with a health-care worker and lasted for several sessions. The strongest effect was found for interventions that lasted 1–3 months^69^. Evidence on interventions similar to ours, i.e., targeting self-efficacy directly via the recall of autobiographical success stories, is indeed mixed: While such an intervention has been found to reduce trauma-related distress and negative affect in torture survivors^71^, in healthy individuals, increased intrusions were observed in the high self-efficacy group^72^. More research is therefore needed to develop and evaluate brief interventions designed to increase self-efficacy in the laboratory context.

Interestingly, our study revealed marked gender differences in the effects of both objective and perceived control. More precisely, interaction effects showed that only in women, controllability protected against increases in negative affect. While the interactions with gender did not reach significance for depression and anxiety, visual inspection of the data indicates that there were also gender differences in the effect of subjective control on these outcomes (see supplementary Figure S2). Apparently, control over stressor exposure (actual and perceived) only decreased negative affective responses in women, but not in men. This contrasts findings in rodents showing that behavioral control alleviates stress effects in male rats, but not females^33^. In female rats, the controlling response failed to activate regions of the PFC that appear to be necessary for the protective effect of control^33^. In a previous study, using a similar fMRI-adapted experimental design, male and female participants showed comparable vmPFC activation associated with control over a stressor^14^, which might explain why women also benefited from control in our study. Interestingly, it seems that males did not fail to profit from control, but rather did not suffer as badly from the lack of control, as they generally reported reduced affective responding. This might be a result of men perceiving more control in general, as indicated by the significant main effect of gender on perceived control. In line with this main effect, control beliefs have been found to be lower in women, possibly owing to lifetime disadvantages in employment opportunities and economic factors, although findings are inconsistent^73^. Indeed, in our sample males and females differed significantly in self-efficacy expectancy (as measured with the General Self-Efficacy Scale) with males displaying higher self-reported self-efficacy expectancy than females (M (SD) male = 31.45 (3.84); M(SD) females = 29.85 (3.91); Welch two-Sample t-test: t(165.99) = 2.72; *p* = 0.007). Our results indicate that specifically women could benefit from stronger perceived controllability which underpins the potential benefits of interventions that boost control beliefs particularly for females.

### Practical implications

Our study can be seen as a proof of concept for the stressor controllability task to investigate differential effects of objective and subjective control over stressors in the laboratory. The results may be valuable for practice, specifically depression therapy, as the need to identify the active components in CBT that are relevant for therapy success is widely acknowledged^74^. Our results suggest that measures that enhance perceived control might be a valuable component to be considered in resilience interventions as well as in therapy for mood disorders.

### Limitations

One of the biggest strengths of the present study is the high variability in perceived control across the two stress groups, but it also represents a potential limitation. The observed disparity between objective and subjective control might stem from the CON group having to endure several seconds of aversive stimulation in each trial. When compared with the NO-STRESS group, participants in the CON group perceived less control and reported more subjective aversiveness, anxiety, negative affect and helplessness, indicating that while they felt more in control than UNCON participants, they experienced at least some amount of helplessness and loss of control. While this allowed us to study different classes of perceived control trajectories, the empirical determination of these classes precluded randomization of individuals and therefore we cannot exclude the influence of other variables. For example, task performance and mean duration of the aversive stimulation differed between the classes and could have contributed to differences in affective reactions. Future studies could therefore adapt the stressor controllability task it in a way that actual control conditions overlap more closely with perceived control. Nevertheless, the task represents an ecologically valid model of an ambiguous situation with respect to controllability and the observed individual differences may reflect different coping styles that provide valuable insights for resilience research.

Another important limitation is the violation of the assumption of equal variances between the experimental groups for helplessness and negative affect. Given that the sample sizes and variances were proportional, this is unlikely to cause a type I error, but it might have reduced power and resulted in less significant results for those outcomes^53^. Nevertheless, the power analysis indicates that the study generally had enough power to detect medium to big effects. As the identification of latent classes based on growth mixture models is a data-driven method, obtaining equal subsamples and variances for the classes is not manageable. However, larger samples generally help with meeting all ANOVA assumptions, underlining the need to acquire large samples when investigating individual differences.

A further limitation is the sample size: While the sample provides sufficient power for the group comparisons as indicated by the power analysis, GMM requires bigger sample sizes. As the method is rather new, specific recommendations do not exist to our knowledge, but especially for more complex models, rather large samples are required. However, in the present study, the model is rather low in complexity due to the low number of classes. A Monte-Carlo study found that a sample of 200 was acceptable to fit a model low in complexity with 4 time points^75^. While our sample is smaller, the higher number of measured time points may in part compensate for smaller sample sizes^75^. Furthermore, the results of the classes largely mirror the results of the group comparisons but with higher effect sizes, indicating that there are consistent effects of actual and perceived control. Nevertheless, the results of the GMM warrant further validation, preferably in a bigger sample.

Finally, the generalizability of the results is limited as we recruited a convenience sample. Many participants were highly educated, 61% of the sample were college students and we only included healthy individuals to identify fundamental mechanisms underlying the influence of stressor controllability on affective responses. Nevertheless, newer approaches to the study of psychopathology like the RDoC framework underline the dimensionality of mental health disturbances in common processes might be responsible for a variety of psychopathological symptoms. Stressor controllability might be such a process and hence, observations found in healthy individuals may still have some relevance for clinical samples^76^. Moreover, emerging adulthood represents a phase of substantial change and mental health problems^77^ and thus the age group investigated here is a vulnerable population that is of interest for resilience research. We found no differences between students (from which only 3 participated for course credits) and non-students, indicating no systematic differences between student and non-student participants. Finally, while the association of locus of control with some mental health outcomes has been found to be dependent on culture, a general, cross-cultural association of locus of control with mental health seems to exist [78]. The present study can thus be seen as a proof-of-concept for the suitability of the stressor controllability task to investigate the processing of controllable und uncontrollable stress in healthy individuals. The generalizability to the general population and the implications for clinical applications are preliminary. Future research should therefore include a more diverse sample and replicate the results in patient groups to investigate whether perceived control also has a protective effect when individuals already show psychopathological symptoms.

## Conclusion

In the present study we demonstrated that, in healthy participants, objective, as well as subjective control over stressors predicts affective responses to those stressors. Individual differences in perceived control were especially relevant for outcomes related to mental health. In particular, trajectories with higher perceived control were associated with lower state depression after a stressful task. Moreover, our results indicate that perceived control might specifically protect women from stress-related affective impairments. These results lend empirical support for theories of stress processing, emotion emergence and resilience that highlight the relevance of controllability appraisals for the stress response^2^, negative emotions^3^ and resilience against psychopathology^4^. In addition to corroborating these theories, the present study translates animal findings on stressor controllability to humans. The employed paradigm may be suited to further investigate stressor controllability effects and inform the development of interventions to enhance perceived control and foster resilience against stress-related disorders. Future work should aim to experimentally manipulate perceived control and to longitudinally assess perceived controllability together with stress-related state variables as well as psychological and biological characteristics in order to explore causal relationships.

## Supplementary Information

Below is the link to the electronic supplementary material.

Supplementary Material 1

## Data Availability

The datasets generated and analyzed during the current study are not publicly available due to the ethical approval and the respective informed consent not including data storage in a public repository. Completely anonymized datasets are available on request from the corresponding author (MW) and after filling out a research agreement.

## Code availability

The R code for all analyses is available at the open science framework under this link: https://osf.io/3xzjr/?view_only=3a41af23d5404a538ee526e8e849c6b7.
