# Supplementary material for: Distinct trajectories of perceived control over aversive stimulation predict affective reactions to stressors over and above objective control
Source: Sci Rep. 2025 Oct 7;15:35009. doi: 10.1038/s41598-025-19958-9 (PMC12504522; doi:10.1038/s41598-025-19958-9)
Supplement: Supplementary file 1 — Supplementary Material 1 [file 41598_2025_19958_MOESM1_ESM.docx]

# Supplementary material for paper “Distinct trajectories of perceived control over aversive stimulation predict affective reactions to stressors over and above objective control”

**Table S1**

*Comparison of affective stress responses of UNCON and UNCON-HSE*

| Variable | UNCON | | | UNCON-HSE | | | *T* | *p* | *d* |
| --- | --- | --- | --- | --- | --- | --- | --- | --- | --- |
|  | *n* | *M* | *SD* | *n* | *M* | *SD* |  |  |  |
| Helplessness | 40 | 4.91 | 1.49 | 40 | 5.42 | 1.48 | -1.54 | .129 | -.34 |
| State depression | 39 | 2.00 | 2.77 | 40 | 2.83 | 3.76 | -1.11 | .270 | -.25 |
| State anxiety | 40 | 1.73 | 4.08 | 40 | 1.03 | 2.95 | 0.88 | .382 | .20 |
| Negative affect | 40 | 1.90 | 2.49 | 39 | 1.72 | 3.42 | 0.27 | .788 | .06 |

*Note.* No significant difference in any affective outcome was observed between the groups experiencing uncontrollable aversive stimulation with (UNCON-HSE) and without a prior self-efficacy intervention (UNCON). One statistical outlier was excluded for state depression (UNCON) and for negative affect (UNCON-HSE).

**Table S2**

*Summary statistics of affective outcomes by group*

| Variable | CON | | UNCON | | NO-STRESS | | *F* | *p* | *η² [CI]* |
| --- | --- | --- | --- | --- | --- | --- | --- | --- | --- |
|  | *n* | *M* | *n* | *M* | *n* | *M* |  |  |  |
| Helplessness | 50 | 4.31^a^ | 80 | 5.16^b^ | 35 | 1.49^c^ | 88.47 | <.001 | .51 [.42,1.0] |
| State depression | 49 | 2.98 | 79 | 2.41 | 37 | 1.54 | 1.84 | .163 | .02 [0.0,1.0] |
| State anxiety | 49 | 1.18^a^ | 80 | 1.38^a^ | 36 | -0.22^b^ | 3.13 | .046 | .04 [0.0,1.0] |
| Negative affect | 45 | 1.38^a^ | 79 | 1.81^a^ | 31 | 0.32^b^ | 4.91 | .008 | .05 [0.0,1.0] |

*Note.* Number of included observations, means and inferential statistics for the affective outcomes. Means with different superscripts significantly differ from each other.
CI: 95%-Confidence Interval of the effect size.

**Figure S1**

*Change in negative affect by stressor controllability group and gender (outliers not removed)*

*
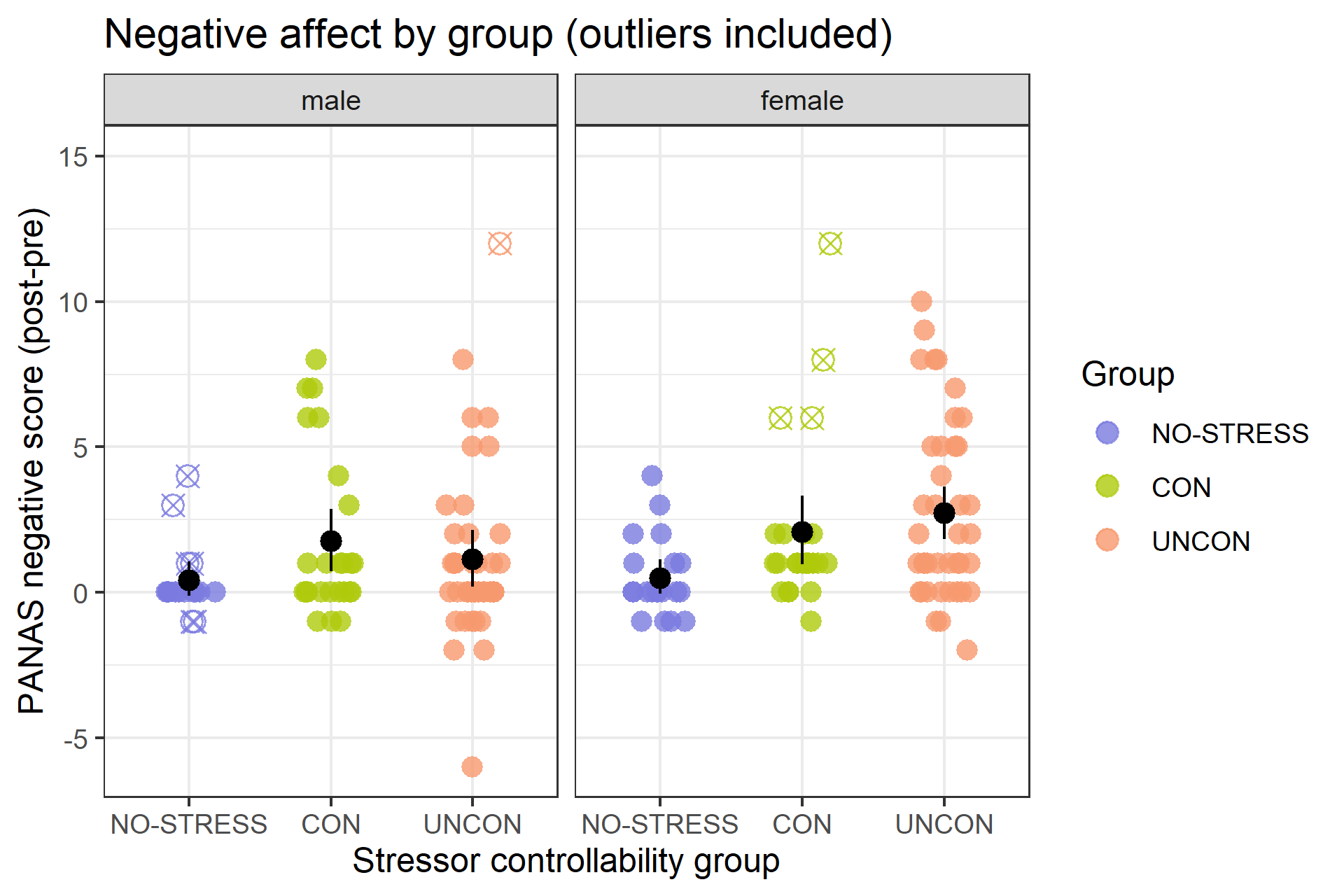
*

*Note.* Mean change in negative affect by group with statistical outliers included. Datapoints classified as outliers marked with crosses. Errorbars denote bootstrapped 95%-confidence intervals for the mean. Removing the 4 outliers in the female CON group reduced the mean of that group so that NO-STRESS and CON do not differ significantly, but CON and UNCON do. With outliers included, there is no significant interaction effect and the contrast NO-STRESS vs. stress groups is significant.

**Table S3**

*Effect of objective control on negative affect with outliers not removed*

| ANOVA table | | | | | | |
| --- | --- | --- | --- | --- | --- | --- |
| Predictor | SS | *df* | MS | *F* | *p* | η^2^ |
| group | 65.44 | 2 | 32.72 | 4.38 | .014* | .05 |
| gender | 31.75 | 1 | 31.75 | 4.25 | .041* | .02 |
| group x gender | 19.21 | 2 | 9.61 | 1.29 | .279 | .02 |
| Error | 1194.59 | 160 | 7.47 |  |  |  |
| Contrasts | | | | | | |
| Name |  |  |  | *T* | *p* |  |
| NO-STRESS vs. stress groups | | | | -2.87 | .005* |  |
| CON vs. UNCON | | | | -0.03 | .975 |  |

*Note.* When not excluding statistical outliers, the interaction of group x gender does not predict negative affect. Only the contrast comparing the no-stress control group (NO-STRESS) to the groups experiencing controllable (CON) and uncontrollable (UNCON) aversive stimulation is significant.
SS = sum of squares, MS = mean square, *p<0.05

**Figure S2**

*Gender differences in the effect of perceived control on affective stress reactions*


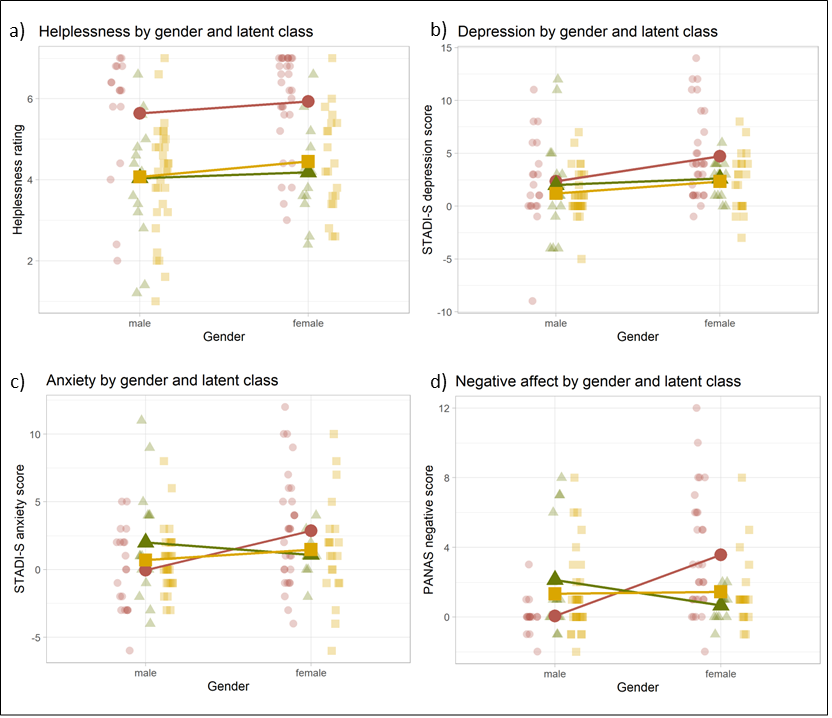


*Note.* While only the interaction effect in the ANOVA for negative affect was significant, visual inspection of the data split by gender indicates that there were also gender differences in the effect of perceived control on depression and anxiety.
